# Autoantibodies are present before the clinical diagnosis of systemic sclerosis

**DOI:** 10.1371/journal.pone.0214202

**Published:** 2019-03-26

**Authors:** Peter D. Burbelo, Sarah M. Gordon, Meryl Waldman, Jess D. Edison, Dustin J. Little, Rodger S. Stitt, Wayne T. Bailey, James B. Hughes, Stephen W. Olson

**Affiliations:** 1 Dental Clinical Research Core, National Institute of Dental and Craniofacial Research, National Institutes of Health, Bethesda, MD, United States of America; 2 Nephrology Department, Walter Reed National Military Medical Center, Bethesda, MD, United States of America; 3 Kidney Disease Branch, National Institute of Diabetes and Digestive and Kidney Diseases, and, National Institutes of Health, Bethesda, MD, United States of America; 4 Rheumatology Department, Walter Reed National Military Medical Center, Bethesda, MD, United States of America; 5 Uniformed Services University of the Health Sciences, Bethesda, MD, United States of America; Medical University of South Carolina, UNITED STATES

## Abstract

Systemic sclerosis (SSc) is a heterogeneous autoimmune disorder associated with vascular dysfunction and fibrotic changes in the skin, vasculature and internal organs. Although serologic abnormalities are an important diagnostic tool for SSc, little is known about whether autoantibodies precede clinical diagnosis. Here we investigated the presence of autoantibodies before SSc diagnosis and assessed whether certain autoantibodies might associate with the future onset of scleroderma renal crisis (SRC), a potentially fatal complication of the disease. Using the Department of Defense Serum Repository, autoantibodies were analyzed from archived, prospectively collected, longitudinal serum samples from sixteen individuals with SRC (SSc/SRC) and thirty cases of SSc without SRC (SSc/no SRC), matched for age, sex, and race. Seventy five percent (12/16) of the SSc/SRC and 40% (12/30) of the SSc/no SRC were seropositive for at least one autoantibody prior to clinical diagnosis (up to 27.1 years earlier, mean = -7.4 years). Although both disease groups demonstrated a heterogeneous immunoreactivity profile against the autoantigen panel, the SSc/SRC subjects showed two enriched clusters with one featuring elevated levels of autoantibodies against Ro52 and/or Ro60 and another with high levels of immunoreactivity against the RNA polymerase complex. Consistent with larger spectrum of immunoreactivity and the elevated levels of autoantibodies in SSc/SRC, the total response against the autoantigen panel from the last time point of the seropositive subjects revealed that the SSc/SRC cohort harbored higher antibody levels (*p* = 0.02) compared to SSc/no SRC. Overall, our findings demonstrate that relevant seropositive autoantibodies often precede the clinical diagnosis of SSc/no SRC and SSc/SRC.

## Introduction

Systemic sclerosis (SSc) is an autoimmune connective tissue disorder associated with significant morbidity characterized by immune activation, vascular abnormalities, and cutaneous and visceral fibrosis [1, 2]. SSc is associated with both a heterogeneous clinical presentation and a diverse autoantibody profile with multiple organs systems affected [3, 4]. Scleroderma renal crisis (SRC) manifests with acute kidney injury and/or accelerated hypertension and is associated with significant morbidity and mortality without appropriate treatment [5, 6]. SRC occurs in 5 to 10 percent of patients with diffuse cutaneous SSc, often within the first four years of disease onset [7–9].

Although the exact disease triggers of SSc are unknown, complex interaction between genes and the environment are thought to be involved. Genome-wide association studies have identified several susceptibility genes related to HLA and immune function [10], but none of these genetic markers are useful for disease screening [11]. In contrast, serologic testing is included as a diagnostic tool for SSc in the ACR/EULAR classification system. Most patients with SSc have circulating autoantibodies directed against one or more of several SSc autoantigens, including topoisomerase I (Topo1), centromere proteins (Cenp-A and Cenp-B), PM/Scl proteins (PM/Scl-100 and PM-SCl-75), RNA polymerase III (RNAP115 and RNAP11), U1-RNP, fibrillarin, Th/To, NOR90, U11/U12 RNP and Ku [12]. These autoantigens are relatively specific for SSc, but individually are only moderately to weakly sensitive. Other autoantibodies targeting Ro52 (also called TRIM21), Ro60, and ribonucleoprotein (Rnp-A) can also be found in SSc, but are not specific to SSc, and are seen in other systemic autoimmune diseases such as SLE, Sjögren’s syndrome, and myositis. Despite the clinical utility of autoantibodies in SSc, the pattern is highly diverse and requires multiple target autoantigens for high sensitivity. A study from Australia found a highly heterogeneous and often non-overlapping autoantibody profile requiring twelve autoantigens to classify most subjects into five major clusters [13]. In this SSc cohort, autoantibodies against Cenp-A/B, Ro52, Topo1 and RNAP III showed the highest seropositivity frequency. Other studies have also shown that certain autoantibodies are associated with clinical subtypes, including the finding that autoantibodies directed against proteins of the RNAP III protein complex are associated with SRC [14–16]. Monitoring changes in the levels of autoantibodies may yield insight into disease progression, but results to date remain inconclusive [17].

Retrospective analysis of several autoimmune diseases including type I diabetes [18], systemic lupus erythematosus [19], rheumatoid arthritis [20] and Sjögren’s syndrome [21], have shown that circulating autoantibodies may be detected years prior to clinical diagnosis of these diseases. Much less is known about the presence and possibly implication of “pre-clinical” autoantibodies in SSc. We undertook this study to determine whether antibodies in SSc/SRC and SSc/no SRC are detectable before clinical diagnosis and whether they associate with disease trajectory or distinct disease manifestations.

Luciferase immunoprecipitations systems (LIPS) is a fluid-phase immunoassay that utilizes luciferase-tagged recombinant antigens to detect antibodies against linear and conformational epitopes of infectious and autoimmune target proteins. We and others have found LIPS to demonstrate high diagnostic performance for detecting autoantibodies in a number of different autoimmune conditions [22] including Sjögren’s syndrome [23], Type I diabetes [24], systemic lupus erythematosus [25], autoimmune gastritis [26], membranous nephropathy [27], and APECED [28, 29]. In several of these studies, LIPS elucidated unique patient autoantibody profiles [23–25, 28] that potentially associated with disease subsets and/or autoimmune symptoms. LIPS with its wide dynamic range of detection and low background has been highly useful for monitoring changes in antibody levels in longitudinal serum samples in both infectious [30] and autoimmune diseases [27]. Here we report our exploratory study profiling autoantibodies from the serum of SSc subjects obtained before clinical disease diagnosis and assess whether unique autoantibody responses might be associated with future onset of SSc/SRC.

## Material and methods

### Ethics statement

The institutional review board of the Walter Reed National Military Medical Center, Bethesda, Maryland approved the protocol (#41833) entitled “Pre-diagnostic longitudinal serology, clinical characteristics, and long-term outcomes for scleroderma renal crisis: a retrospective case control study”. Informed consent was waived by the Walter Reed National Military Medical Center for the testing of coded, stored serum samples from the Department of Defense (DOD) Serum Repository (DoDSR) and for the review of clinical records due to the innocuous nature of the study and the potential to acquire important medical information. To protect the privacy of the scleroderma patients, their names and unique personal information were not recorded or released. Analysis of the serum autoantibodies of the cohort at the NIH (#13309) was approved through the Office of Human Subject Research.

### SSc/SRC and SSc/no SRC subject selection and serum samples

Administered by the Army Medical Surveillance Activity starting in 1985, the DoDSR has banked serum samples from U.S Armed Forces personnel starting at entry into the military. Samples are then collected longitudinally every 1–2 years as well as before and after deployments. In addition, all active duty military personnel have a full medical assessment at the onset of their service time, yearly throughout their career, before and after deployments, and at retirement. Currently over 50 million serum samples are stored at -30°C. Despite the large number of stored serum samples, not all personnel have banked serum, some have limited time points, and serum may not have been collected at the time of autoimmune diagnosis.

**Fig 1:**
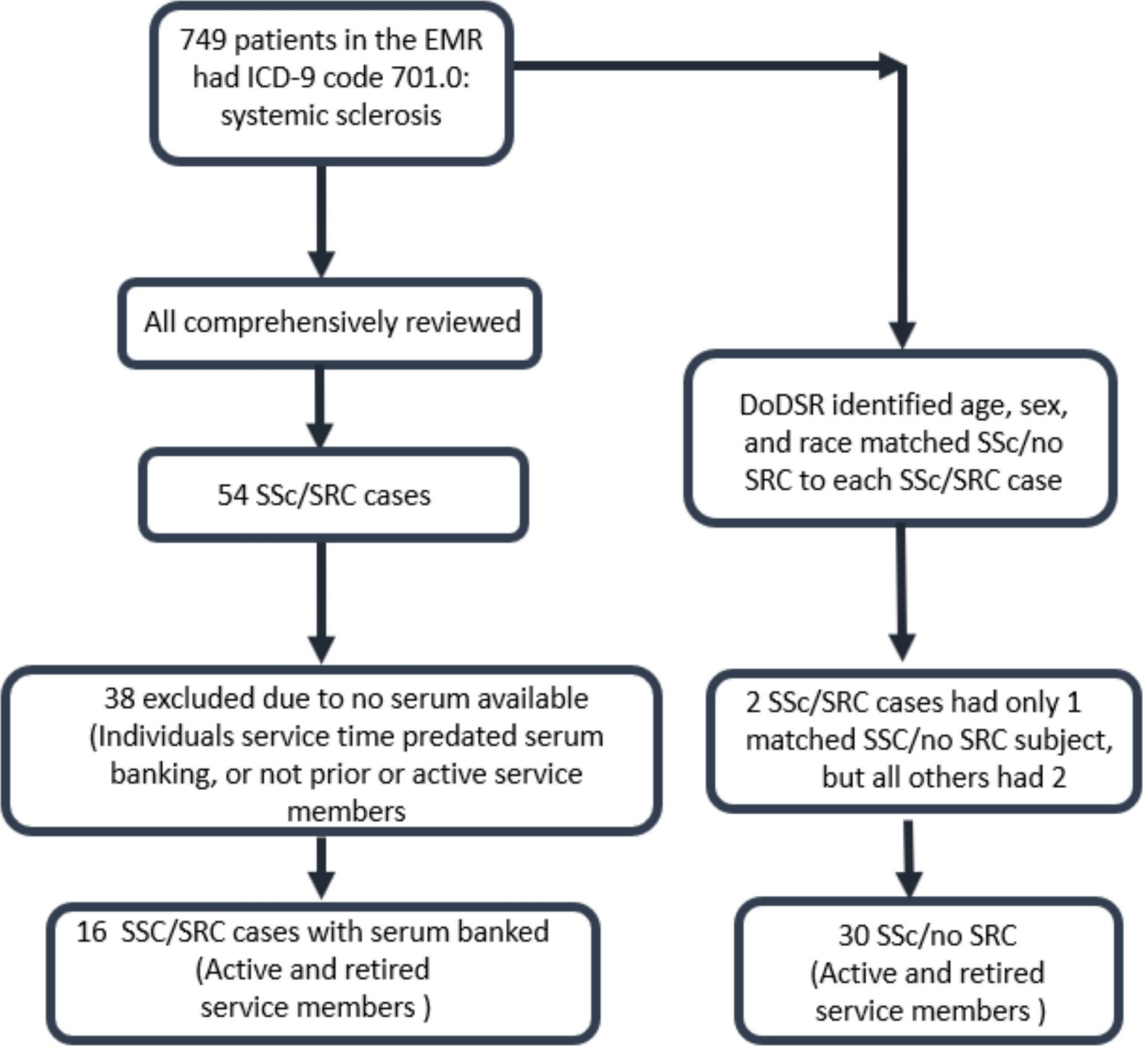
Flow-chart for selection of the SSc/SRC and SSc/no SRC cases. As described in the material and methods, screening of the military electronic medical records between 2005–2016 for Systemic Sclerosis was initially preformed. Following comprehensive review, 54 cases of SS/SRC were identified, of which only 16 had available serum samples. Thirty additional SSc/no SRC cases were then identified that were matched for age, gender and race with the SS/SRC group.

Here we performed a retrospective case control study to assess the preclinical autoantibody profile of subjects who subsequently developed SSc/SRC and SSc/no SRC. The authors of this paper who participated in establishing inclusion and exclusion criteria include rheumatologists who have previous experience diagnosing and treating scleroderma at Walter Reed National Military Medical Center. The flowchart for the selection process of the final cohort is shown in Fig 1. By screening the military electronic medical records between 2005–2016 for the International Classification of Diseases, 9th Revision, Clinical Modification (ICD-9-CM) code for Systemic Sclerosis (710.1), 749 SSc cases were identified. All electronic medical records were then reviewed for evidence of SRC. SRC was defined by at least one of the following criteria in the absence of another clinical explanation for AKI and/or hypertensive emergency: 1). Acute kidney injury requiring renal replacement therapy (RRT); 2). A doubling of serum creatinine; 3). A 50% rise in serum creatinine with new onset hypertension (blood pressure greater than or equal to 140/90 mmHg); and 4). Hypertensive urgency or emergency defined by an abrupt onset of BP ≥180/110 mmHg requiring hospitalization or evidence of end organ damage. Fifty-four cases met the criteria for SRC. However, thirty-eight of the SSc/SRC cases did not have serum banked in the DoDSR because some cases were dependents of active duty members, and some were retirees who had left the military prior to the systematic banking of serum. This left sixteen cases, in which the background clinical data from the electronic medical record was collected and analyzed (Fig 1). The following data were collected for the SSc/SRC cases when present: age, sex, race, year of SSc diagnosis, age at SSc diagnosis, year of SRC diagnosis, pulmonary fibrosis (pulmonologist documentation or chest computed tomography), pulmonary hypertension (pulmonologist or cardiologist documentation or evidence on echocardiogram), cardiac involvement (pericarditis, pericardial effusion, or new and otherwise unexplained heart failure, documented by cardiology consultation or echocardiogram), Raynaud’s phenomenon (RP), gastrointestinal involvement (gastroesophageal reflux disease or esophageal dysmotility), prior corticosteroid use, and other immunosuppression therapy, as well as the presence of anti-nuclear (ANA), anti-centromere (ACA), anti-topoisomerase I (Scl-70), anti-RNAPOL3 antibody, Ro, La, and anti-U3 RNP antibodies (Table 1). Each of these cases had stable blood pressure, blood pressure medication regiment and serum creatinine from 2 years prior to SSc diagnosis up until the acute SRC event. This was a subgroup of cases from a previously reported large retrospective cohort study (32). The DoDSR then attempted to match two SSc/no SRC subjects for each SSc/SRC case by gender, race and age (within ±1 year), and age of the serum samples (Fig 1). To maximize diagnostic specificity by the DoDSR, SSc/no SRC was defined as at least one inpatient or three separate ambulatory ICD-9 coded encounters for SSc without any ICD-9 codes for kidney involvement or hypertensive urgency/emergency. Because these matching SSc/no SRC disease controls were selected and de-identified by the DoDSR per protocol, background clinical data could not be collected.

**Table 1:** Clinical characteristics of SSc cohort.

	SSc/SRC Cases (n = 16)	SSc/no SRC (n = 30)
Race		
Black	63% (10/16)	60% (18/30)
White	31% (6/16)	40% (12/30)
Sex		
Female	44% (7/16)	43% (13/30)
Male	56% (9/16)	57% (17/30)
Age at diagnosis	43.8 (+/-11.8)	34.9 (+/- 8)
Time followed after SSc diagnosis (years)	7 (6–9)	NA
Time between SSc diagnosis and SSc/SRC (years)	2 (1–5)	NA
PF	56% (9/16)	NA
PHTN	38% (6/16)	NA
Cardiac	13% (2/16)	NA
Esophageal dysmotility	81% (13/16)	NA
Raynaud’s	94% (15/16)	NA
DU	38% (6/16)	NA
Cancer	6% (1/16)	NA
Prednisone use	56% (9/16)	NA
IST	31% (5/16)	NA
ANA	88% (14/16)	NA
ANA Speckled pattern	46% (5/11)	NA
Anti-SCL-70	14% (2/14)	NA
Anti-RNAPOL3	25% (2/8)	NA
Anti-U3-RNP	9% (1/11)	NA
SSA	31% (4/13)	NA
SSB	8% (1/13)	NA
Anti-centromere	0% (0/10)	NA

Available clinical data is shown for the SSc/SRC group. SSc/no SRC cases were de-identified which prevented full data collection by chart review. SSc/no SRC = Systemic sclerosis with no SRC, SSC/SRC = scleroderma renal crisis, PF = pulmonary fibrosis, PHTN = pulmonary hypertension, DU = Digital ulcerations, IST = immunosuppressive therapy excluding prednisone, ANA = anti-nucleolar autoantibody, C3/C4 = low complement 3 and 4 value, SCL-70 = Anti-topo I autoantibody positivity, Anti-RNApol3 = RNA polymerase III autoantibody positivity, U3-RNP = fibrillarin autoantibody positivity, SSA = SSA autoantibody positivity, SSB = SSB autoantibody positivity, and NA = not available.

In total, 16 SSc/SRC cases and 30 matching SSc/no SRC controls were studied (Fig 1). For most subjects, three 0.5 mL serum samples from different time points were available for analysis corresponding to the oldest sample, the second to last sample before diagnosis and the most recent sample before diagnosis. In a few disease controls, a serum sample was tested after SSc diagnosis if that sample better approximated the age of the serum sample of the matched SRC case. A total of 121 samples from 46 different individuals were identified and the cohort of blinded serum samples was then provided to the researcher (P.D.B.) at the National Institutes of Health.

### Luciferase-antigen fusion proteins and LIPS autoantibody testing

LIPS, a powerful method employing light-emitting proteins [22], was utilized to measure and monitor autoantibodies. Seven previously described luciferase-antigens were employed including Ro52 (N-terminus), Ro60, La, Rnp-A, Sm-D3, PM/Scl-100 and Jo-1 and their performance and cut-off values have been described in other conditions [23, 25, 31]. Five new autoantigen fusions were generated including for Cenp-A, PM/Scl-75, POLR3A (also called RNAP115), POLR3K (also called RNAP11) and Topo1. DNA sequence analysis was used to confirm the integrity of the five new plasmid constructs.

These twelve different luciferase-antigen constructs were transfected into Cos1 and the cell lysates containing the light emitting fusion proteins were harvested [32]. Briefly, the lysates were centrifuged twice at 13,000 x g and the supernatants collected and used immediately or stored frozen. A tube luminometer (20/20 from Turner Scientific) was used with coelenterazine or NanoGlow substrate mix (Promega, Madison, WI) to determine the luciferase activity of each autoantigen lysate in light units (LU).

For testing, serum samples were first aliquoted into master, deep well microtiter plates by diluting serum 1:10 in buffer A (50 mM Tris, pH 7.5, 100 mM NaCl, 5 mM MgCl_2_, 1% Triton X-100 and 0.001% bromophenol red). For LIPS autoantibody analysis against a specific autoantigen target, 40 μl of buffer A, 10 μl of diluted sera from the master plate (1 μl equivalent), and 1 × 10^7^ light units (LU) of luciferase-antigen cell extract was put into to each well of a 96-well microtiter plate and incubated for 1 hour at room temperature. The entire 100 μl antigen-serum antibody reaction mixture was then pipetted into a filter plate (Millipore Sigma) containing 5 μl of a 30% suspension of protein A/G beads. After further incubation for 1 hours with shaking, the antibody complexes bound to the protein A/G beads were washed eight times with buffer A and twice with PBS using a plate washer. After the final wash, LU were measured in a Berthold LB 960 Centro microplate luminometer (Berthold Technologies, Bad Wilbad, Germany) using coelenterazine for *Renilla* luciferase fusion proteins or NanoGlow (Promega) substrate mix for nanoluciferase fusions. Based on pre-determined cut-off values for each autoantigen, seropositivity status of samples from the SSc cohort was determined before the codes were broken. Lastly, analysis of additional blood donor controls (n = 30) were evaluated retrospectively for determining diagnostic specificity of the five newly developed SSc LIPS tests (Cenp-A, Scl75, POLR3A, POLR3K and Topo1) and ensure they were not over-estimating seropositivity. For this LIPS autoantibody assessment, the blood donor controls were tested side-by-side the lowest seropositive SSc samples identified for each of the 5 autoantigens.

### Data analysis

GraphPad Prism software (San Diego, CA) was used to plot the autoantibody levels in the different subjects and for statistical analysis. A colored heatmap was used to compare the relative autoantibody levels between the different subjects for each of twelve different antigens. This cutoff value was first subtracted from the autoantibody levels for each antibody-sera pair, and the resulting value was divided by the corresponding cut-off value to yield a relative level of the autoantibody, which was then color coded from yellow to dark black. Mann-Whitney *U*-tests were used to compare autoantibody levels between the SSc/SRC and SSc/no SRC groups.

## Results

### Clinical characteristics of the cohort

Due to funding limitations and the need for proof of concept that prediagnostic autoantibodies exist in this complex disease, a retrospective study analyzed 121 archived longitudinal serum samples from 46 scleroderma subjects with SSc/SRC (n = 16) and SSc/no SRC (n = 30), but without disease controls. As summarized in Table 1, a majority of the subjects in cohort were black (~ 60%) and male (~56%). The mean age of diagnosis was 43.8 ± 11.88 years in the SSc/SRC subjects and 34.9 ± 8.0 years in the SSc/no SRC subjects. The median onset of SRC after SSc diagnosis was 0.5 year. As shown in Table 1, the most common clinical feature in the SSc/SRC group was Raynaud’s (94%) followed by pulmonary fibrosis (56%). Available autoantibody data on the SSc/SRC subjects is also shown.

### Seropositive autoantibodies are often present years before SSc/no SRC and SSC/SRC onset

**Fig 2:**
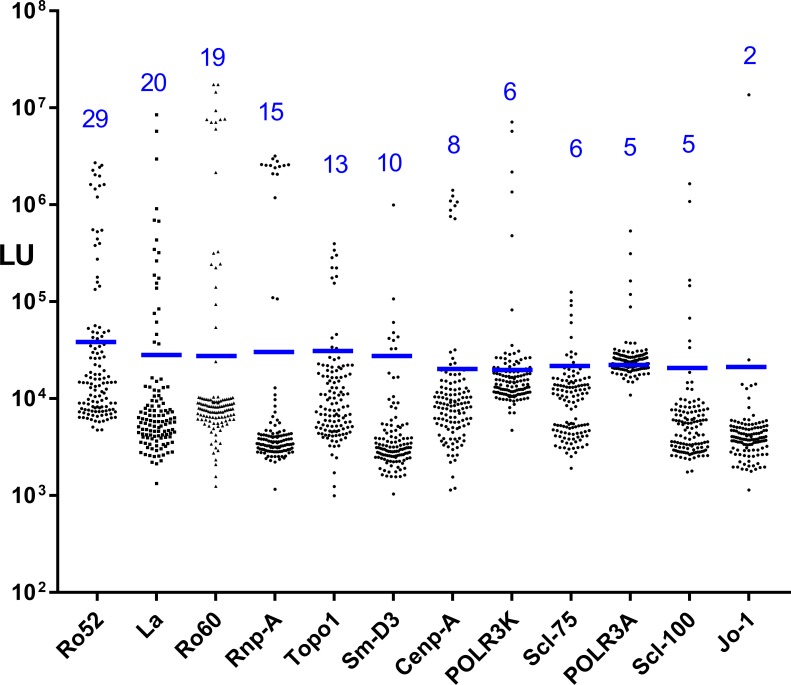
Serum autoantibody levels in the SSc/SRC and SSc/no SRC cohort. Autoantibody measurements were made by LIPS against the 12 autoantigens in the 121 blinded serum samples corresponding to a total of 16 SRC and 30 SSc/no SRC cases. The Y axis reflects the antibody levels in LU determined by LIPS. The blue line is the cut-off value for each antigen. As shown by the numbers in blue, the most prevalent autoantibodies were against Ro52, while the least common were against the Jo-1 autoantigen.

Based on the established autoantibody heterogeneity in SSc, serum autoantibodies were measured against a panel of autoantigens. For this study the LIPS technology was used and included seven previously described LIPS autoantibody tests. In addition, five new LIPS tests were developed for this study that were relatively-specific for SSc, including against Cenp-A, PM/Scl-75, POLR3K, POLR3A, and Topo1 (Table 2). While the Cenp-A autoantigen comprised only the known antigenic N-terminal region [33], the four other new SSc target autoantigens utilized full-length proteins fused to luciferase. This panel of twelve LIPS autoantibody tests were then used to evaluate the 121 serum samples of the SSc cohort in a blinded fashion. Cut-off values were assigned for each antigen and seropositivity status for each serum-antigen pair was established before un-blinding occurred. LIPS profiling demonstrated a large dynamic range of autoantibody detection in subject sera, often differing by 1000-fold (Fig 2). However, using defined cut-off values for each autoantigen, only a subset of samples was seropositive for any given autoantigen. The number of autoantibody seropositive serum samples detected was as follows: 29 for Ro52, 20 for La, 19 for Ro60, 15 for Rnp-A, 13 for Topo1, 10 for Sm-D3, 8 for Cenp-A, 6 for POLR3K, 6 for PM/Scl-75, 5 for POLR3A, 5 for PM/Scl-100 and 2 for Jo-1.

**Table 2:** Characteristics and specificity of luciferase-SSc autoantigen constructs.

SSc Autoantigen	Amino acids	Autoantigen location	Luciferase	Specificity^1^
**Cenp-A**	1–35 (fragment)	N-terminus	Nano	100%
**Scl-75**	1–439 (full-length)	N-terminus	Nano	100%
**POLR3A**	1–1390 (full-length)	N-terminus	Nano	100%
**POLR3K**	1–107 (full-length)	C-terminus	Renilla	100%
**Topo1**	1–765 (full-length)	C-terminus	Renilla	100%

^1^ From testing 30 blood donor controls with internal SSc controls.

Further evidence for the validity of some of our findings and consistent with a published report [13], was the findings of co-positivity of 5 of 6 serum samples against the two structurally different subunits of RNAP III and co-positivity for 4 of the 6 sera against the two distinct PM/Scl autoantigens. Because the SSc cohort did not include non-SSc, control serum samples and to show that the new five new uncharacterized SSc LIPS tests were not over-estimating seropositivity, an additional 30 blood donor controls were tested. For this calibrated analysis, we retrospectively tested the blood donor controls side-by-side with the lowest seropositive SSc samples for each autoantigen and found each of the LIPS tests had 100% specificity (**Table 2**). Together these findings suggest that autoantibodies against the five new SSc target proteins detected in the SSc cohort potentially represent true seropositives against these target proteins.

Following sample unblinding and further analysis, 75% (12/16) of the SSc/SRC and 40% (12/30) of the SSc/no SRC subjects harbored at least one positive autoantibody sample before diagnosis. It is important to point out that in the SSc/SRC cases only 44% (7/16) had SSc-associated autoantibodies. Using the Mann-Whitney *U* test, the SSc/SRC group showed a statistically significant higher frequency of seropositivity than the SSc/no SRC group (p = 0.03). Both groups showed a similar mean time for the earliest detectable seropositive sample before diagnosis (SSc/SRC = -9.6 years and SSc/no SRC = -5.7 years). The earliest detection of autoantibody in one SSc/SRC patient was 27.1 years prior to clinical diagnosis and remained positive over time before diagnosis.

**Fig 3:**
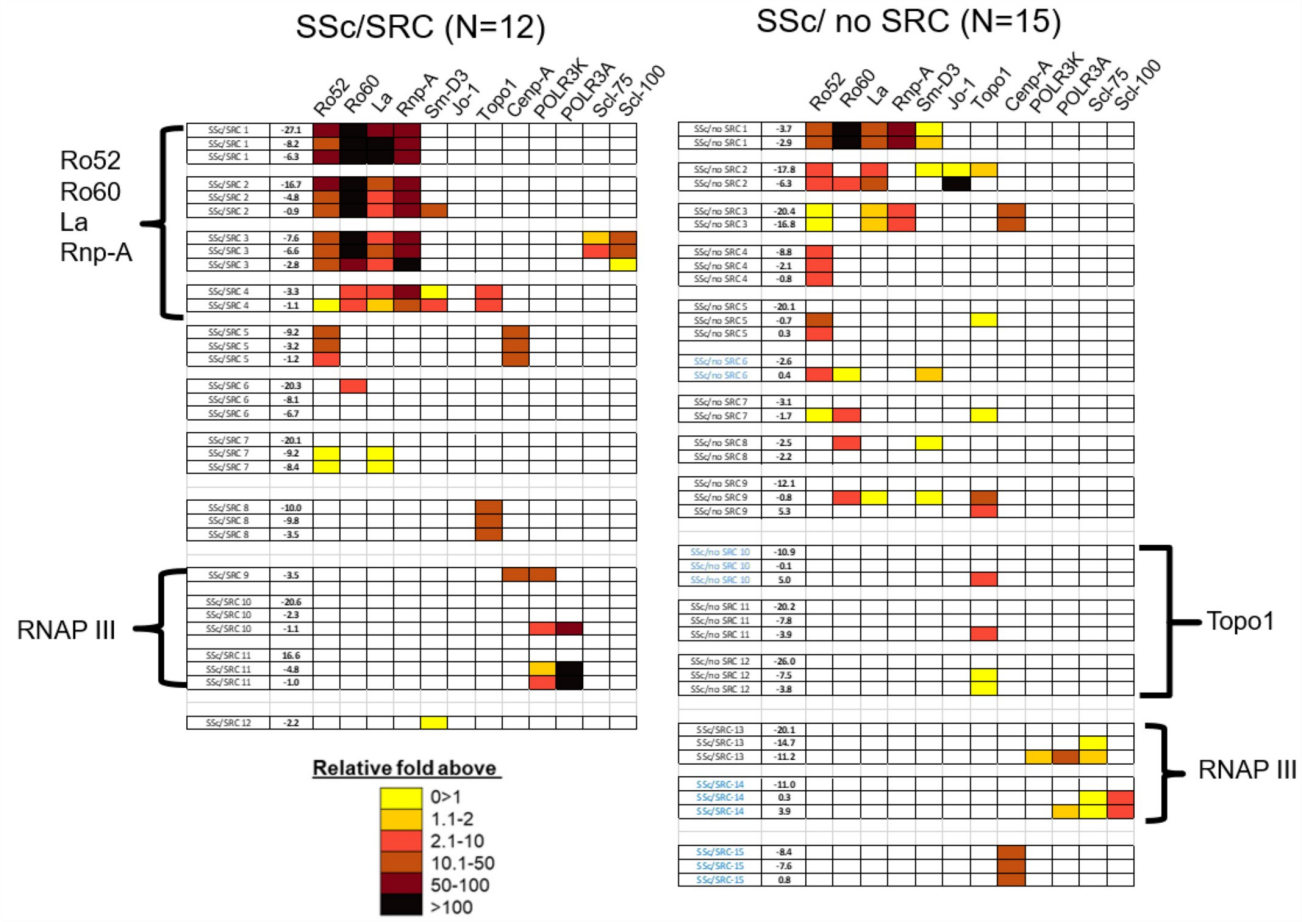
Heatmap analysis of autoantibodies in the SSc/SRC and SSc/no SRC cases. Heatmap analysis of autoantibody responses are shown in the 12 seropositive SSc/SRC and 15 seropositive SSc/no SRC subjects. For each case, the time in years before (-) or after (+) initial systemic sclerosis diagnosis is denoted in the column on the left. Each group of rows represents the autoantibody profile in a single case, in which the blue colored codes represent SSc cases with autoantibodies detected after diagnosis. Color coding denotes relative antibody levels above the baseline cut-off value and the clear boxes represent seronegative responses with the autoantigens in a given subject. As shown by the key, seropositive autoantibody levels in the subjects ranged from low levels (yellow) to extremely high autoantibody levels (black). Based on the patterns that emerged, the SSc/no SRC and SSc/SRC subjects were then manually segregated into three autoantibody clusters for Ro6o/Ro52/La/Rnp-A, Topo1 and RNAP III.

Based on the known clinical and serologic heterogeneity in scleroderma along with the relatively small sample size of our cohort, the prediagnostic autoantibody data is best interpreted as multiple case series or subgroups with similar autoantibody profiles. A heat map was generated to facilitate the identification of these subgroups (Fig 3). This analysis revealed several pre-clinical autoantibody clusters that may help elucidate multiple pathophysiologic pathways. The seropositive SSc/SRC cases (n = 12) had two distinct antibody clusters. One cluster of 4 (25%) cases showed highly elevated levels of Ro52, Ro60, Rnp-A, and La autoantibodies during the preclinical phase prior to diagnosis. A second cluster of three SSc/SRC patients showed elevation of autoantibodies against RNAP III subunits nearer to diagnosis.

**Fig 4:**
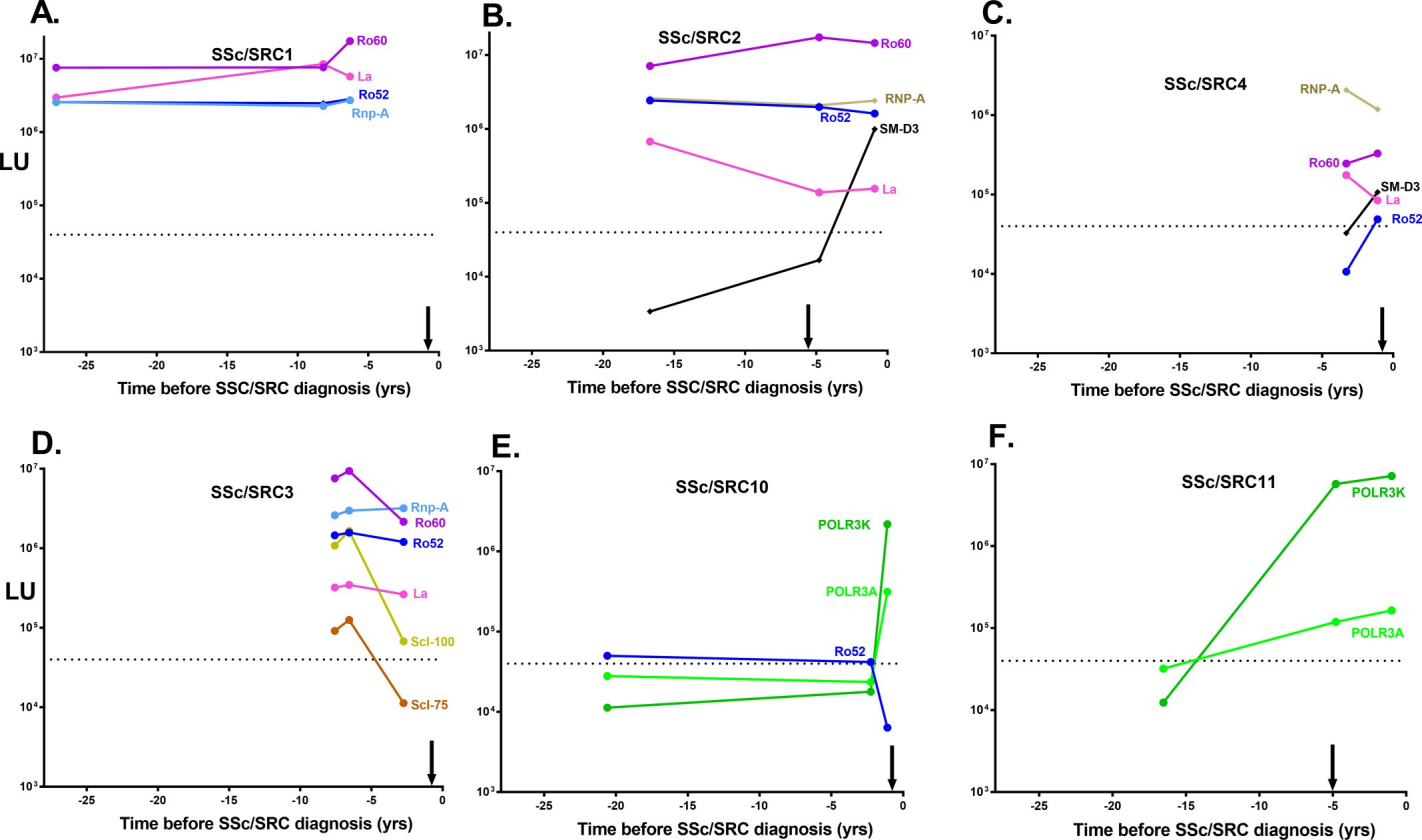
Representative autoantibody profiles seen in SSc/SRC cases before disease diagnosis. Representative plots illustrating autoantibody levels in six SSc/SRC subjects before disease diagnosis. The X-axis denotes the time in years before diagnosis of SSc/SRC (time 0). The approximate time of SSc/no SRC diagnosis preceded SSc/SRC and is denoted by the black vertical arrow. The left Y axis represents the autoantibody levels in LU and the dotted line represent the approximate cut-off value for the autoantigens.

Fig 4 shows representative more detailed, longitudinal line plots of autoantibody profiles from individual SSc/SRC patients in the clusters. In four cases, Ro60 autoantibodies were detected in the earliest available serum samples (i.e. -27.1, -16.7, -7.6, and -3.3 years) and remained persistently elevated in these subjects leading up to SSc/SRC diagnosis (Fig 4A–4D**)**. High levels of autoantibodies against Ro52, Rnp-A and La autoantigens were also observed in these SSc/SRC subjects in a similar pattern as the Ro60 autoantibodies. In two other SSc/SRC subjects, autoantibodies were found against RNAP III subunits between -1.1 and -4.8 years prior to SRC diagnosis (Fig 4E and Fig 4F). In one of the subjects, autoantibodies against two components of RNAP III, POLR3A and POLR3K, rose 13- and 122-fold, respectively from a seronegative status only -1.1 years before diagnosis suggesting a rapid evolution of the autoantibody response before SSc/SRC manifests (Fig 4F).

**Fig 5:**
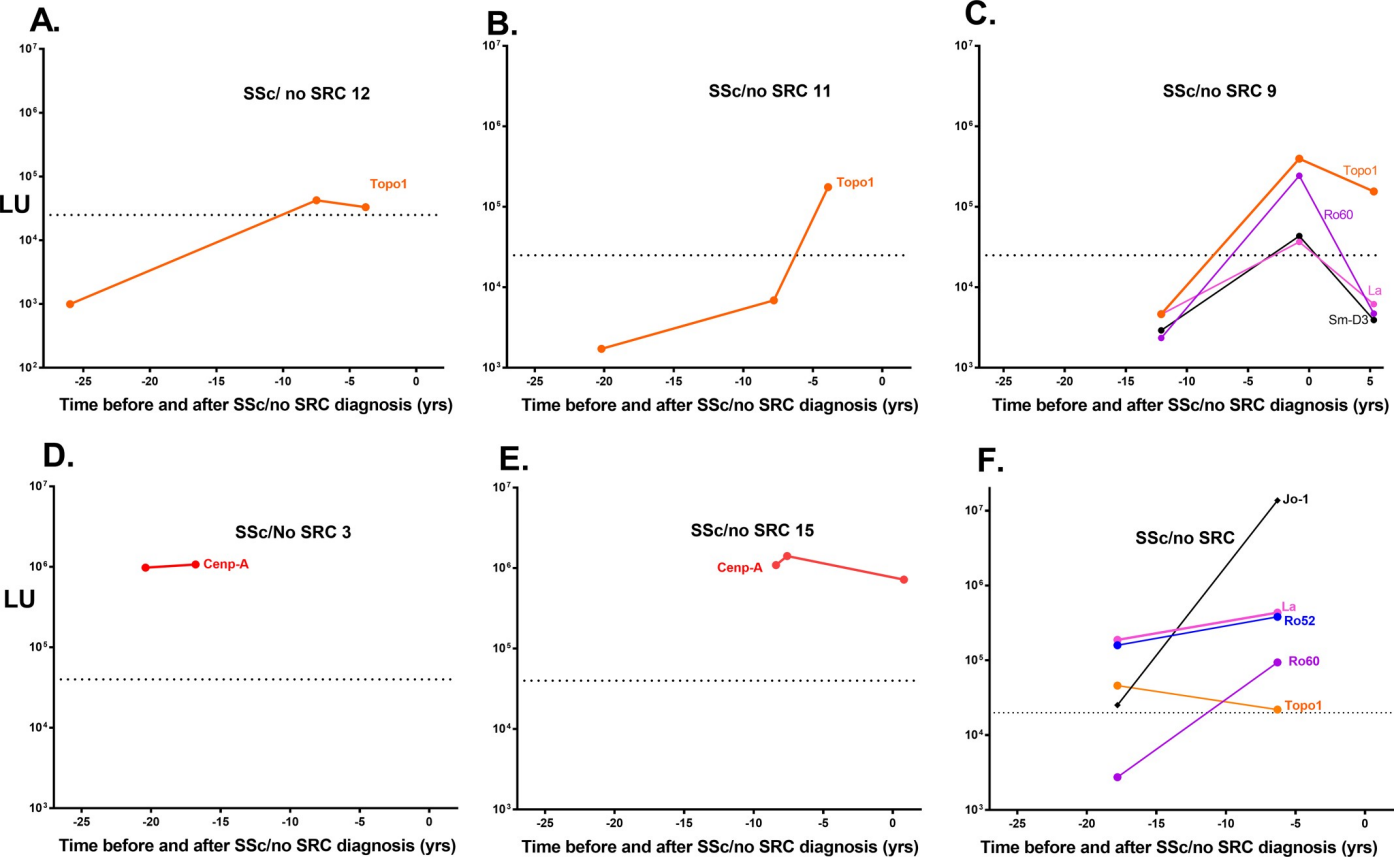
Representative autoantibody profiles seen before diagnosis of SSc/no SRC. Representative plots illustrating autoantibody levels in the six SSc/ no SRC subjects before diagnosis of the disease. The X-axis denotes the time in years before diagnosis. The left Y axis represents the scale of the autoantibody levels in LU and the dotted line represent the cut-off values for the antigens. All seropositive antibody responses against the autoantigen panel are shown.

There were also notable autoantibody clusters in the SSc/no SRC subjects (Fig 3). As shown in the longitudinal line plots for representative SSc/no SRC cases, Topo1 autoantibodies in three subjects rose prior to diagnosis (Fig 5A–5C). Two other SSc/no SRC subjects had significantly elevated Cenp-A antibody levels that persisted from the earliest available preclinical sample (-20.4 and -8.4 years) to the last sample (Fig 5D and Fig 5E). Lastly, one SSc/no SRC subject had multiple autoantibodies present before diagnosis including against Ro52, Ro60, La, Topo1 and Jo-1 autoantigens (Fig 5F). Interestingly, the anti-Jo-1 autoantibody levels in this subject, an autoantibody usually associated with myositis, rose dramatically over time when approaching SSc diagnosis.

Autoantibody clustering may be at least partially explained by race. Ro60 or Ro52 autoantibody was present before SSc diagnosis in 35% (16/46) of SSc/SRC cases. Prediagnostic Ro60 autoantibody was only elevated in blacks [43% (12/28) vs. 0% (0/18), p<0.001]. While there was a similar percentage of black SSc/SRC cases and black SSc/no SRC controls with elevated prediagnostic Ro60 autoantibody [50% (5/10) vs. 39% (7/18), p = 0.69], more black cases with SSc/SRC cases had a Ro60 autoantibody levels greater than 10 times normal, [50% (5/10) vs. 6% (1/18), p = 0.01]. Black SSc/SRC cases were also more likely to have consistently elevated prediagnostic Ro60 autoantibody over time in addition to simultaneously elevated La autoantibody (>30,000 LU), and RNP autoantibody (>30,000 LU) than black SSc/no SRC disease controls [40% (4/10) vs. 6% (1/18), p = 0.04 for both]. When present in SSc/SRC cases, Ro60 autoantibody was greater than 10 times normal in the oldest index sample in all 5 SSc/SRC cases (26.1, 20.3, 16.7, 6.6, and 2.3 years before diagnosis). The one SSc/no SRC disease control with a Ro60 autoantibody level greater than 30 times normal had stable and normal longitudinal prediagnostic Topo-1 autoantibody levels. Prediagnostic Ro52 autoantibody was elevated in the setting of a normal Ro60 autoantibody for 1 SSc/SRC case and 3 SSc/no SRC disease controls, all of whom were white. Ro52 autoantibody was normal in the setting of an elevated Ro60 autoantibody in one case and four disease controls, all of whom were black.

**Fig 6:**
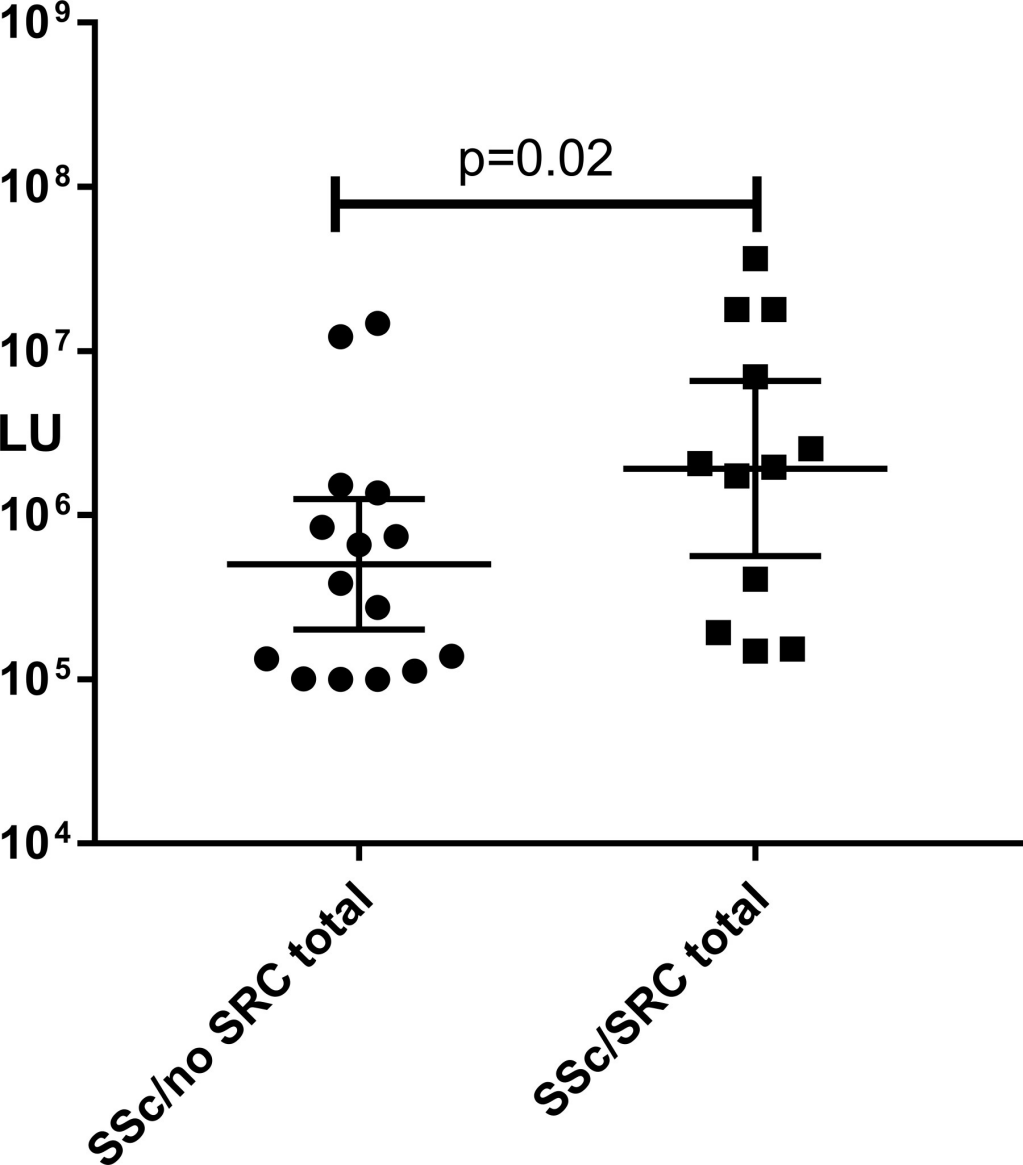
Higher autoantibody levels in SSc/SCR compared to SSc/no SRC cases. The scatter plot graphs represent the total antibody response in individual seropositive SSc/SRC and SSc/no SRC cases. This analysis was accomplished by summing up the autoantibody values against the twelve autoantigens from the last time point of the seropositive subjects. The *P* values were calculated using the Mann-Whitney *U* test.

In addition to highlighting the complex autoantibody profiles, the Fig 3 heatmap also suggested that SSc/SRC cases had a greater number of autoantibodies and at higher levels than the SSc/no SRC controls. More SSc/SRC cases had greater than one prediagnostic autoantibody compared to SSc/no SRC [50% (8/16) vs. 20% (6/30), p = 0.04]. In addition, more SSc/SRC cases harbored very high levels of prediagnostic autoantibodies greater than 1,000,000 LU than SSc/no SRC [50% (8/16) vs. 13% (4/30), p = 0.01 respectively]. Finally, we examined the total antibody response in the SSc/SRC cases versus SSc/no SRC controls by summing up the autoantibody values against the twelve autoantigens from the last time point of the seropositive subjects. As shown in Fig 6, the SSc/SRC group showed higher antibody levels (*p* = 0.02) compared to the SSc/no SRC disease controls. While these results suggest that the breadth and magnitude of autoantibody levels are different between the two groups, SSc/SRC group, we acknowledge that other unknown differences in clinical characteristics between the SSc/no SRC group and the SSc/SRC group may confound our results.

## Discussion

To our knowledge this is the first study to show that autoantibodies are present for years prior to clinical diagnosis of SSc/SRC and SSc/no SRC and confirm that pre-clinical autoantibody profiles are as diverse as the known autoantibody profiles seen after disease onset. Using the LIPS technology, approximately 52% of the SSc/no SRC and SSc/SRC subjects had detectable autoantibodies before diagnosis. These values may underestimate the true percentages as some subjects only had serum samples available more than 2 years before diagnosis. Moreover, we also likely underestimated the prevalence of autoantibodies as we did not test for other relevant SSc autoantibodies including Cenp-B, PDGF receptor, fibrillarin, NOR90 and Th/To [13, 17]. The autoantibody trends suggest that the Ro52, Ro60 and Cenp-A autoantibodies are elevated decades before disease manifestations and remain elevated approaching diagnosis, while RNAP-III, Topo1, and anti-Jo-1 autoantibodies rise over time and become elevated a faster pace prior to clinical disease. Lastly, autoantibodies were present in a few subjects at early time points and then disappeared. Excluding technical issues, it is formally possible there are fluctuations in autoantibodies during early stages of the disease before clinical diagnosis and further studies are needed to explore this possibility.

While historically many studies have utilized immunoprecipitation using ^35^S-methionine labeled cell extracts to quantitate autoantibodies in SSc and other autoimmune diseases, this approach has been supplanted by antigen-specific immunoassay, employing recombinant autoantigens including labeling with radioactive by *in vitro* transcription/translation, ELISA, line immunoblot, LIPS, and other technologies [12, 22]. The LIPS technology, which overexpresses target autoantigens linked to luciferase, is particularly attractive because it does not require radioactivity, allows direct quantitation of autoantibodies levels against defined autoantigens and has similar or higher sensitivity and specificity then established approaches [22]. A previous study analyzing thirteen recombinant autoantigens by line immunoblot assay in a cohort of Australian SSc subjects also identified unique autoantibody clusters [13]. This cohort of SSc subjects was comprised mainly of white women and the most prevalent autoantibodies were against Cenp-A/Cenp-B, followed by Ro52, Topo1 and then RNAP III [13]. In contrast, our clustering analysis with the DOD cohort found the most common immunoreactivity was directed against Ro52 and Ro60 followed by Topo1 and RNAP III and there was a paucity of immunoreactivity against Cenp-A. Consistent with our findings, multiple other studies have found a high frequency of Ro52, and to a lesser extent Ro60 autoantibodies, in SSc cases from diverse geographical locations including Canada [34, 35], Spain [36], Germany [37] and China [38]. In one study by Fritzler and colleagues, Ro52 autoantibodies were detected as the second most prevalent autoantibody in a Canadian SSc cohort and was a marker on interstitial lung disease and overlap syndrome [34]. Consistent with our findings, another study specifically examining racial differences in SSc, SSA seropositivity was found to be more common in blacks at SSc diagnosis with a rate of 25% seropositivity [39]. Thus, the high prevalence of African Americans, gender and disease subtype differences in our study may have accounted for the atypical SSc autoantibody profile. Prediagnostic Ro60 antibody strongly associates with the African-American race. The prevalence of prediagnostic Ro60 and Ro52 autoantibody and association with SRC is consistent with recent publication reporting that seropositivity against SSA (comprising autoantibodies against both Ro60 and Ro52 as a single test) at SSc diagnosis is associated with future risk of SSc/SRC [40]. Previous literature also supports the observation that Ro52 and Ro60 seropositivity is one of the earliest markers of risk for future manifestation of other autoimmune diseases. Arbuckle et al found that SSA seropositivity was present for the longest duration before the onset of SLE (mean time before = -9.4 years) and were present in the earliest available samples in 64% of cases [19]. While there was no breakdown of specific Ro52 and Ro60 autoantibody seropositivity by race, this study found a higher percent of pre-diagnostic SSA autoantibody seropositivity than traditionally reported at SLE diagnosis (47%) which may have been explained by the disproportionately high percentage of black subjects (62%) in the study population derived from the DOD [19]. The summation of our findings and the literature suggests that subclinical Ro52 and Ro60 autoantibodies may contribute early in the pathophysiology of autoimmunity in certain subpopulations. Since Ro52 and Ro60 are known to be involved in several aspects of pathogen clearance [41, 42] and innate immunity [43], respectively, one interpretation of these findings is that the appearance of these autoantibodies before clinical diagnosis reflects some inherent altered immune dysfunction in these individuals, potentially triggered by pathogen exposure. Further studies using molecular and serological analysis of the longitudinal serum samples from SSc/SRC and SSc/no SRC cases, particularly at the time of autoantibody seroconversion may shed light on the exact infectious agent, if any, that might drive the loss of tolerance to self-proteins.

Another finding of our study was the potential link of high levels autoantibodies against RNAP III with the onset of SRC. Although previous cross-sectional studies have detected an association of RNAP III autoantibodies with existing SRC [44–47], our findings suggest that a burst in high levels of RNAP III autoantibodies occurs in a subset of patients shortly before (i.e. within approx. 1 year) the onset of renal crisis. Little is known about the mechanism by which RNAP III autoantibodies is associated and/or potentially participates in the pathophysiology leading to SRC. Lastly, the elevated pre-diagnostic RNAP III autoantibodies were not elevated in the same SSc/SRC cases with elevated Ro52 and Ro60 autoantibodies supporting the idea that there are multiple pathogenic pathways to the development of SRC.

In conclusion, our study of SSc/SRC and SSc/no SRC cases demonstrates that seropositive autoantibodies occur years before clinical diagnosis in approximately 52% of the subjects, which is likely an underestimation. It is also important to point out that our study has several limitations and caveats. First, like other autoimmune studies documenting autoantibodies from retrospective biobank studies [19–21], it is possible that unrecognized symptoms associated with the presence of autoantibodies were present before official diagnosis. Second, there is no universal definition of SRC which makes comparisons within the literature difficult. We chose a rigorous and transparent definition which was as stringent if not more so than other studies. A consensus definition is needed [36]. We were unable to account for tendon rubs and skin thickening due to inconsistent and incomplete reports in the medical record. Despite efforts to match for age, there were differences in some covariates between the two groups. The small study samples size inherent to the rarity of SSc/SRC precluded multivariate analysis to account for potential confounding variables. Therefore, we cannot rule out significant differences between the study groups for other clinical characteristics that may have contributed to or accounted for the observed autoantibody associations with SSc/SRC. We did not have access to the full clinical background information of the SSc/no SRC disease controls provided by the DoDSR because of their de-identification requirements. This prevented potentially insightful findings about other organ system involvement in SSc and associations with autoantibody profiles. Additionally, the disease may be difficult to diagnose and thus the timing of the diagnosis in the DoDSR system may not accurately reflect onset of symptoms. Direct clinical selection of the SSc/no SRC disease controls would have facilitated a more comprehensive understanding of background clinical details, but the likely poor matching of age, race, sex, and particularly age of serum sample would have introduced greater limitations. Our study cohort was skewed more toward male gender and black race than previous studies and may not be extrapolated to other populations (i.e. females) that are more often afflicted by SSc. Despite these limitations, the novel findings of the study can instruct future research. The presence of diverse prediagnostic autoantibody profiles in the current study with a small sample size of SSc/SRC and SSc/no SRC cases justifies a follow up prediagnostic autoantibody study. Optimally this future study would include a large SSc cohort supported by detailed background clinical characteristics and an expanded autoantibody profile to include the addition of other SSC-specific autoantibody targets [12], such as Cenp-B, fibrillarin, U1 RNP, NOR 90, Th/To, U11/U12 RNP, PDGFR, and Ku.
